# Metabolome and Its Mechanism Profiling in the Synergistic Toxic Effects Induced by Co-Exposure of Tenuazonic Acid and Patulin in Caco-2 Cells

**DOI:** 10.3390/toxins16070319

**Published:** 2024-07-15

**Authors:** Yuxian Qin, Hongyuan Zhou, Yulian Yang, Ting Guo, Ying Zhou, Yuhao Zhang, Liang Ma

**Affiliations:** 1 College of Food Science, Southwest University, Chongqing 400715, China; qyx20000420@163.com (Y.Q.); zhouhy@swu.edu.cn (H.Z.); linjukyang@163.com (Y.Y.); guoting0615@126.com (T.G.); zhouyying@swu.edu.cn (Y.Z.); zhy1203@163.com (Y.Z.); 2 Chongqing Key Laboratory of Speciality Food Co-Built by Sichuan and Chongqing, Chongqing 400715, China; 3 Key Laboratory of Quality and Safety Control of Citrus Fruits, Ministry of Agriculture and Rural Affairs, Southwest University, Chongqing 400715, China; 4 Key Laboratory of Luminescence Analysis and Molecular Sensing, Southwest University, Ministry of Education, Chongqing 400715, China; 5 Key Laboratory of Condiment Supervision Technology for State Market Regulation, Chongqing 401121, China

**Keywords:** mycotoxins, Caco-2 cells, combined toxicity, metabolomics, signaling pathways

## Abstract

Tenuazonic acid (TeA), usually found in cereals, fruits, vegetables, oil crops, and their products, was classified as one of the highest public health problems by EFSA as early as 2011, but it has still not been regulated by legislation due to the limited toxicological profile. Moreover, it has been reported that the coexistence of TeA and patulin (PAT) has been found in certain agricultural products; however, there are no available data about the combined toxicity. Considering that the gastrointestinal tract is the physiological barrier of the body, it would be the first target site at which exogenous substances interact with the body. Thus, we assessed the combined toxicity (cell viability, ROS, CAT, and ATP) in Caco-2 cells using mathematical modeling (Chou-Talalay) and explored mechanisms using non-targeted metabolomics and molecular biology methods. It revealed that the co-exposure of TeA + PAT (12.5 μg/mL + 0.5 μg/mL) can induce enhanced toxic effects and more severe oxidative stress. Mechanistically, the lipid and amino acid metabolisms and PI3K/AKT/FOXO signaling pathways were mainly involved in the TeA + PAT-induced synergistic toxic effects. Our study not only enriches the scientific basis for the development of regulatory policies but also provides potential targets and treatment options for alleviating toxicities.

## 1. Introduction

Mycotoxins, as a class of fungal secondary metabolites of fungi, are widespread in nature and can produce various toxic effects on human and animal health throughout the food chain. Food intake is the main way for the body to be exposed to mycotoxins. As the first barrier to prevent exogenous chemicals from entering the body to exert toxic effects, the intestinal tract faces more severe challenges than other organs and its homeostasis plays an important role in maintaining health [1]. Thus, the Caco-2 cell line was selected to be the in vitro model in the current study because of its similar morphology and function to human intestinal epithelial cells, which provides the possibility to better evaluate the local toxicity caused by exogenous chemicals entering the intestine [2].

Tenuazonic acid (TeA), as an emerging mycotoxin, is the most contaminated mycotoxin that is produced by *Alternaria* after naturally infecting crops [3,4]. In 2011, the European Food Safety Authority (EFSA) conducted the first safety assessment of *Alternaria* toxins in food and feed and recommended that TeA should be listed as one of the highest public health concerns based on the available dietary exposure data, which showed that the average chronic dietary exposure to *Alternaria* toxins of 30,788 European adults (18–65 years old) exceeded the corresponding TTC value [4,5]. Generally, TeA was often found in cereals, tomatoes, and their products, and also sometimes detected in bell peppers, grapes, apples, citrus, figs, wine, sunflower seeds, and beer, and its contamination level in dry fruit samples could reach up to 81,592 μg/kg [6,7,8,9,10,11,12,13]. Furthermore, it is also worth noting that TeA has a high contamination rate in infant food [9]. In 15 European countries surveyed by the EFSA, the detection rate of TeA in infant food was as high as 96.51% [5]. Moreover, owing to immature organ development, poor detoxification ability, high food intake per unit body weight, and grain-based food as the main source of energy and nutrition, infants and young children may have a higher risk of TeA dietary exposure than adults. However, studies on the toxic effects of TeA are still very limited. It has been reported that TeA can cause vomiting, diarrhea, gastrointestinal bleeding, weight loss, the inhibition of ribosomal activity, the obstruction of protein synthesis and release, and could even be related to the occurrence of esophageal, but its related targets and molecular mechanisms are still unclear [4,6,14,15]. Therefore, due to the lack of toxicological data on TeA, the limit standards and regulations in food and feed have not been promulgated, even though TeA has become a global concern.

More importantly, the environment is always teeming with various contaminants that can produce toxic effects on living organisms in the form of mixtures in real scenarios [16]. Patulin (PAT), produced by *Penicillium*, *Aspergillus*, *Byssochlamys*, etc., usually contaminates fruits and vegetables, especially apples and their products [17]. It has been found that the coexistence rate of TeA and PAT in apples was as high as 46.43% and the coexistence of TeA and PAT was also detected in apple juice, dried fruit (raisins, dried apricots, and dates), and nut samples [10,18]. As a traditional mycotoxin, PAT has demonstrated that it has hepatotoxicity, nephrotoxicity, neurotoxicity, immunotoxicity, and carcinogenicity and can induce nausea, vomiting, intestinal bleeding, and duodenal lesions, which present a gastrointestinal toxicity phenotype similar to that of TeA to some extent [19,20]. It has been reported that the combination of beauvericin (BEA), PAT, and sterigmatocystin (STE) can increase the toxicological effects of CHO-K1 cells [21]. Similarly, a mixture of alternariol monomethyl-ether (AME) and TeA resulted in stronger cytotoxicity on HepG2 cells, and a synergistic effect of PAT and ochratoxin A (OTA) on gastrointestinal barrier integrity at low levels was also found in Caco-2 cells [22,23]. However, studies on the combined toxic effects of TeA and PAT are very limited, except for the only report from our laboratory demonstrating that synergic toxic effects can be induced by the co-exposure of TeA and PAT to *C. elegans* [24]. Therefore, further investigations on the specific mechanism of toxicity should be conducted. Here, the combined toxic effects of the mixture of TeA + PAT were evaluated in our study using the CI evaluation model established by Chou-Talalay, which has the advantages of providing a visual assessment of the interactions between chemicals and not being affected by the interactions between the tested chemicals, aiming to enrich the toxicology profiles of TeA and/or PAT [25,26].

At present, studies on the assessment of combined toxicity often use phenotypic or biochemical indicators as the endpoint but the mechanisms are still unclear. Metabolomics, as a systematic biological technology developed in recent years, can clarify the changes in all metabolites with molecular weights between 50 Da and 1500 kDa, such as lipids, sugars, amino acids, nucleic acids, steroids, etc., in organisms or cells after stimulation with exogenous chemicals by qualitative (non-targeted) or quantitative (targeted) detection of the end products of cell metabolism in order to reflect the effect of exogenous toxic substances on the biological function of cells and provide clues for exploring corresponding mechanisms. Recently, metabolomics has been widely applied to further study the evaluation of toxicity, mechanisms of action, and biomarker identification of exogenous chemicals [27]. In this study, the combined toxicity of TeA and PAT was predicted by the CI evaluation model (Chou-Talalay) based on Caco-2 cells and its mechanisms were further studied using metabolomics and molecular biology technologies, which aimed to systematically and comprehensively explore the toxicological data of co-exposure of TeA and PAT, enrich the theoretical basis for further improvement of relevant regulations and standards, and provide potential targets and treatment options for alleviating the corresponding toxicities.

## 2. Results

### 2.1. Combined Toxicity Evaluation Based on Cell Viability

**Figure 1:**
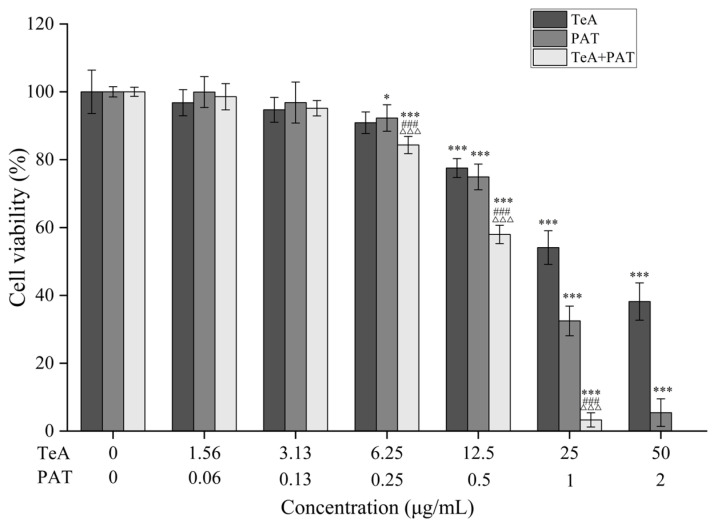
Effects of TeA and PAT alone and their binary mixtures on the viability of Caco-2 cells. Cells were exposed to the corresponding concentrations of mycotoxins for 48 h and then the viability was determined by CCK-8, among which no viable cell was detected at the highest concentration of TeA + PAT group. Data are expressed as mean values ± SD of independent experiments (*n* = 3). * and *** represent significant difference (*p* < 0.05) or extremely significant difference (*p* < 0.001) from the control group; ^###^ and ^△△△^ represent extremely significant difference (*p* < 0.001) to the TeA- and PAT-alone exposure groups, respectively.

Pilot studies on dose responses were conducted to investigate the respective toxic effects of TeA and PAT on Caco-2 cells. A dose-dependent reduction in cell viability was observed, and the IC_50_ values of the two mycotoxins were also obtained using SPSS with probit regression. Specifically, the IC_50_ values and corresponding 95% confidence interval (CI) values of TeA and PAT were 37.42 (33.87~41.78) μg/mL and 0.96 (0.88~1.03) μg/mL, respectively. Therefore, the highest experimental concentration of TeA and PAT can be set as 50 μg/mL and 2 μg/mL, respectively, with a concentration ratio of 25:1. Then, a two-fold gradient dilution method was used to set five concentration gradients to explore the combined toxic effect of TeA and PAT on Caco-2 cell viability. As the results show (Figure 1), the cell viability of all the tested groups decreased with an increase in the exposed concentrations in a dose-response manner. Specifically, the co-exposure of TeA and PAT at lower concentrations (TeA < 3.13 μg/mL, PAT < 0.13 μg/mL) did not significantly reduce the cell viability when compared with the single exposure of each mycotoxin. Nevertheless, as the concentration increased, the inhibition of the TeA + PAT-treated group was significantly higher than that of the TeA-/PAT-treated groups. When exposed to 6.25 μg/mL of TeA or 0.25 μg/mL of PAT alone, the cell viability of each group was higher than 90%, while when exposed to the mixture of TeA and PAT, the cell viability dropped to 84.33% (*p* < 0.001). Notably, at the highest concentrations of TeA (50 μg/mL) and PAT (2 μg/mL), no viable cell was detected in the TeA + PAT-treated group. Then, the type of interaction between TeA and PAT was assessed using the Chou–Talalay method shown in Table 1 [20]. As a result, the additive effect was displayed in the mixture of TeA + PAT at the lower concentration (IC_25_) while synergistic effects were revealed at the medium-to-higher concentrations (IC_50_~IC_75_). The difference in interaction types is affected by many factors, such as exposure concentrations, the experimental model, biotransformation, the target organ, the mode of action, etc. [28].

### 2.2. Cellular Oxidative Stress Assessment

**Figure 2:**
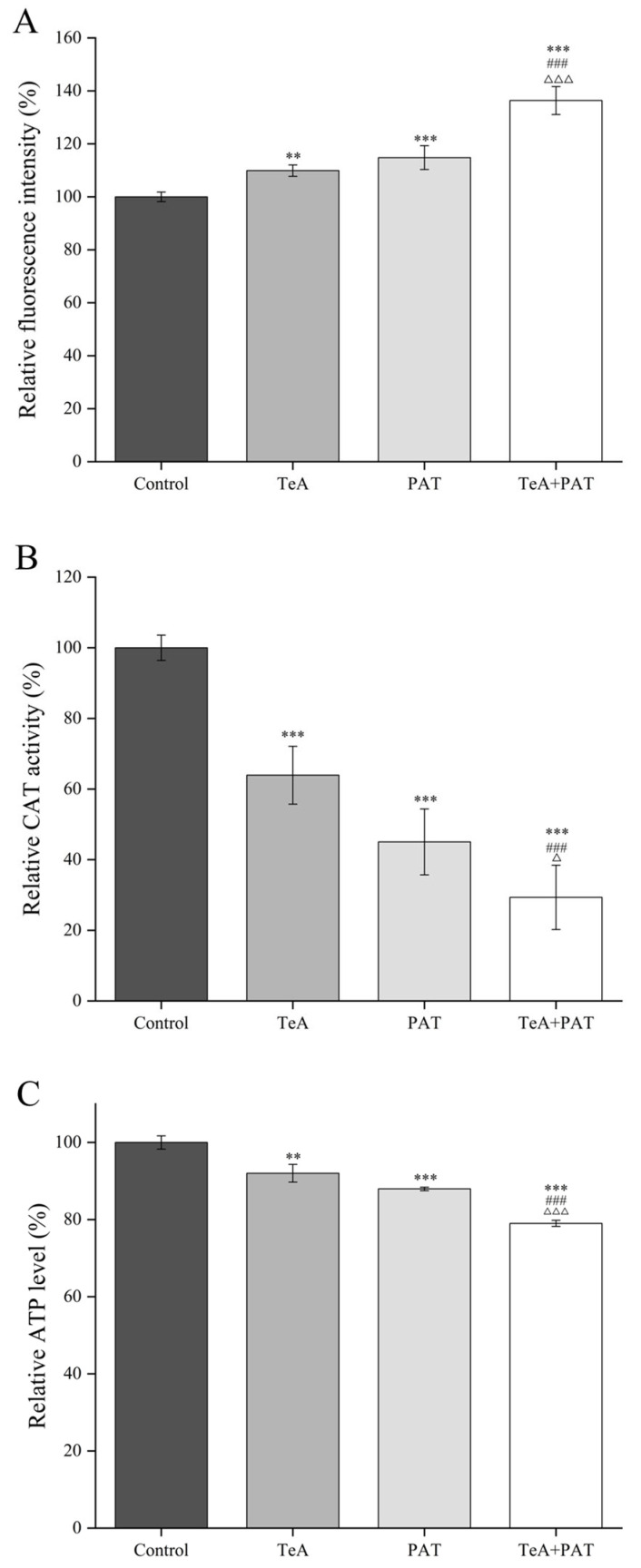
Effects of TeA PAT alone and its binary exposure (48 h) on ROS (**A**), CAT (**B**), and ATP (**C**) of Caco-2 cells. The concentrations of TeA, PAT, and TeA + PAT are 12.5, 0.5, and 12.5 + 0.5 μg/mL, respectively. Data are expressed as mean values ± SD of independent experiments (*n* = 3). ^△^ represent significant difference (*p* < 0.05) compared to the PAT-alone exposure groups; ** and *** represent extremely significant difference (*p* < 0.01, *p* < 0.001) compared to the control group; ^###^ and ^△△△^ represent extremely significant difference (*p* < 0.001) to the TeA- and PAT-alone exposure groups, respectively.

The concentrations of TeA and PAT of 12.5 μg/mL and 0.5 μg/mL, respectively, can induce a significant reduction in the cell viability as well as synergistic toxic effects on the co-exposed cells, so the above concentrations were selected to conduct the following experiments. The degree of ROS accumulation could be determined by staining the cells with a DCFH-DA fluorescent probe. As shown in Figure 2A, the fluorescence intensity was 109.89% and 114.81%, respectively, when the Caco-2 cells were exposed to TeA or PAT alone, while the fluorescence intensity of the TeA + PAT-exposed cells increased to 136.37% (*p* < 0.001), indicating that the co-exposure of TeA and PAT can cause a surge in intracellular ROS content and affect the REDOX balance in cells. Meanwhile, CAT, as an indicator reflecting the antioxidant capacity, was significantly reduced to 29.34% in the cells exposed to the mixture of TeA + PAT when compared to the control, which was also much lower than that of the TeA- or PAT-exposed groups (63.92% or 45.03%) (Figure 2B). Moreover, ATP is an essential substance to maintain normal physiological activities, which is involved in the synthesis and metabolism of a variety of nutrients. As displayed in Figure 2C, the ATP levels were 92.02% and 87.96%, respectively, when Caco-2 cells were treated with TeA or PAT alone. However, once the Caco-2 cells were treated with the mixture of TeA + PAT, the ATP levels were reduced to 79.02% (*p* < 0.001), showing that the co-exposure of TeA and PAT can significantly reduce the ATP content of the cells.

### 2.3. Cellular Metabolites Analysis

**Figure 3:**
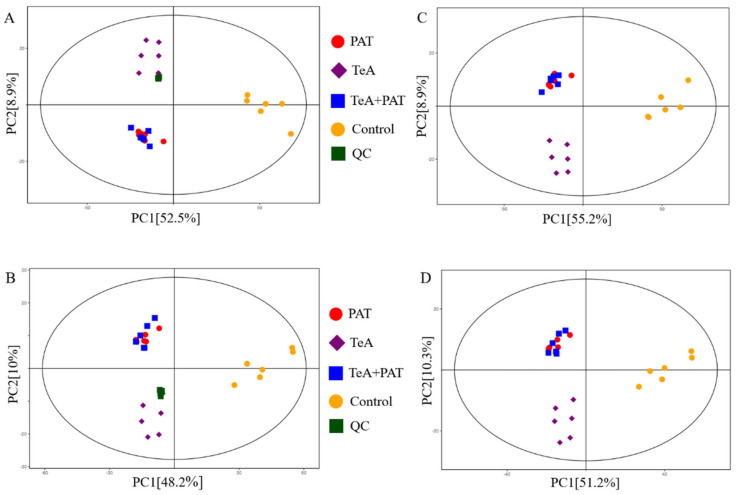
PCA scores of QC and experimental samples. (**A**) QC samples in positive ion mode; (**B**) QC samples in negative ion mode, (**C**) experimental samples in negative e ion mode; (**D**) experimental samples in negative ion mode.

To further explore the effects of TeA, PAT alone, and their binary exposure on cellular metabolic function, UHPLC-MS/MS non-targeted metabolomics was performed to explore the important metabolic pathways and mechanism of the synergistic toxicity of TeA and PAT. The metabolic profiles of the control, TeA, PAT, TeA + PAT, and QC groups (*n* = 6) were analyzed to assess the stability and reliability of the experimental method. The results showed that all samples were in the 95% CI in both positive (POS) and negative (NEG) ion modes with good clustering of QC samples, indicating good instrument stability and method repeatability, as well as accurate and stable test data in this study (Figure 3A,B). Meanwhile, as shown in Figure 3C,D, the TeA, PAT, and TeA + PAT groups were prominently separated from the control group, indicating that the metabolic mode of the cells in the experimental group and the control group had markedly changed. Moreover, obvious deviations were also observed between the TeA-treated group and the other two mycotoxin-treated groups, while partial overlap was found between the PAT-treated group and the TeA + PAT-treated group, suggesting that PAT might play a dominant role in exerting the combined toxic effect of TeA + PAT and TeA enhanced the overall toxicity to some extent. In this study, 9822 and 9386 metabolites were detected in POS and NEG modes, respectively. Thus, to improve the analysis coverage, POS mode was selected for the subsequent data analysis.

In addition, the OPLS-DA model revealed noticeable differences in the distribution of metabolites between the TeA-, PAT-, and TeA + PAT-treated groups and the control group with R^2^Y ≈ 1 and Q^2^ = 0.924, 0.939, and 0.941, respectively, which demonstrated that the OPLS-DA model was well predictive and reliable (Figure S1). According to the screening criteria of variable importance in projection (VIP) > 1 and *p* < 0.05, 522 significantly different metabolites (SDMs) were identified and analyzed. The number and classification of SDMs in Caco-2 cells exposed to TeA, PAT, and TeA + PAT, respectively, are shown in Figure S2. Specifically, 336 SDMs (316 up-regulated and 20 down-regulated) were found in the TeA-exposed group, among which 64.76% of them were lipids and lipid-like molecules, 9.34% were organic acids and derivatives, 7.23% were organoheterocyclic compounds, and 5.42% were organic oxygen compounds. As for the PAT-treated group, 338 SDMs (302 up-regulated and 36 down-regulated) were detected with 57.44% lipids and lipid-like molecules, 11.31% organic acids and derivatives, 7.44% organoheterocyclic compounds, and 6. 58% organic oxygen compounds. Furthermore, in the TeA + PAT-treated group, 326 SDMs (294 up-regulated and 32 down-regulated) were discovered, of which lipids and lipid-like molecules accounted for 58.95%, organic acids and derivatives for 11.73%, organoheterocyclic compounds for 7.41%, and organic oxygen compounds for 6.48%. Overall, after exposure to TeA, PAT, and TeA + PAT, the metabolisms of lipids and lipid-like molecules in Caco-2 cells were mainly affected.

Then, a Venn diagram analysis was performed on the differential metabolites in each group to summarize the differential and overlapping SDMs among all the experimental groups (Figure 3A). There were 289 and 296 identical differential metabolites between TeA + PAT co-exposure and TeA or PAT exposure alone, respectively. Furthermore, we also observed 274 of the same SDMs among TeA, PAT, and TeA + PAT groups with 25, 11, and 6 different SDMs, respectively, indicating that TeA and PAT may have similar toxic modes of action to regulate cellular metabolisms.

**Figure 4:**
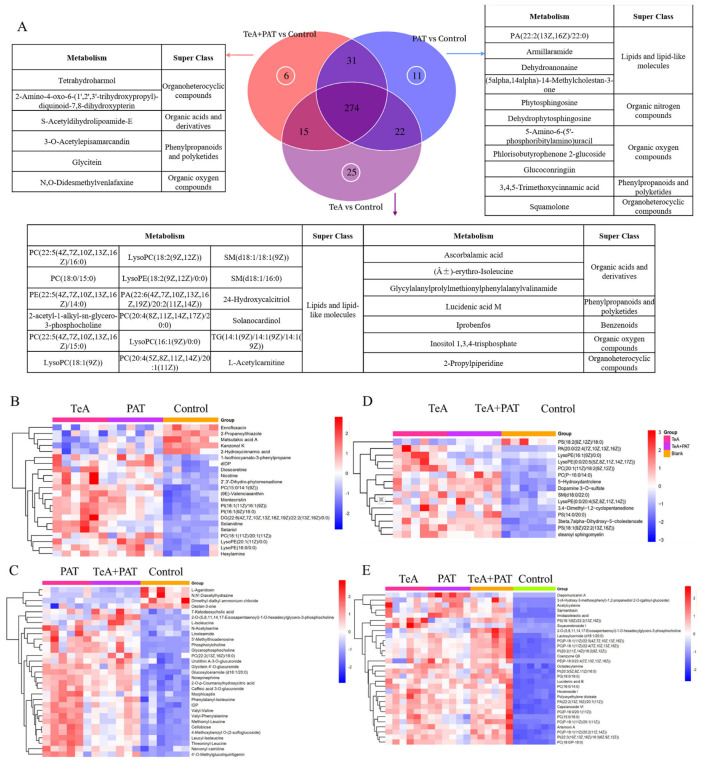
Metabolic profiles of TeA-, PAT-, and TeA + PAT-treated Caco-2 cells. (**A**) Venn diagram of differential metabolites in each experimental group; (**B**–**E**) the heatmaps generated by hierarchical clustering of the common altered metabolites of cells exposed to TeA and PAT, TeA and TeA + PAT, PAT and TeA + PAT, and TeA and PAT and TeA + PAT, respectively.

Furthermore, cluster heatmap analysis was performed on the common SDMs of the TeA-, PAT-, and TeA + PAT-exposed groups to explore the changing trends. As shown in Figure 4A, the TeA and PAT groups had 22 identical differential metabolites, different from those of the TeA + PAT group, which were mainly glycerophospholipids and a small number of steroids, fatty acids, fatty acyl, aromatic compounds, organoheterocyclic compounds, sesterterpenes, organic acids, ketones, isoflavones, etc. Compared with the control group, the expression of five SDMs, in both TeA and PAT groups, was down-regulated while seventeen SDMs were upregulated (Figure 4B). Except for in the PAT group, there were 15 identical SDMs in TeA and TeA + PAT, mainly glycerophospholipids and a small number of bile acids, sphingolipids, organic acids, ketones, etc., among which the expression of 1 SDM was down-regulated and 14 SDMs were up-regulated (Figure 4C). Meanwhile, 31 identical SDMs of PAT and TeA + PAT groups were obtained, which were mainly amino acids, glycerophospholipids, and a small number of sugars, nucleosides, organic nitrogen compounds, bile acids, neurotransmitters, sphingomyelins, lignin, flavonoids, and isoflavones (Figure 4D). As for the three experimental groups, 274 identical SDMs in total were found. Then, according to the corresponding VIP scores and fold-change (FC) values (Table S2), we screened out the top 30 identical SDMs, mainly glycerophospholipids, amino acids, and a small number of aromatic compounds, fatty acids, fatty acids, sphingolipids, quinones, terpenoids, etc. Compared with the control group, the expression of the 30 identical SDMs was up-regulated, and the FC of phosphatidylcholine PC (15:0/16:0) was the highest.

### 2.4. Cellular Metabolic Pathways Analysis

**Figure 5:**
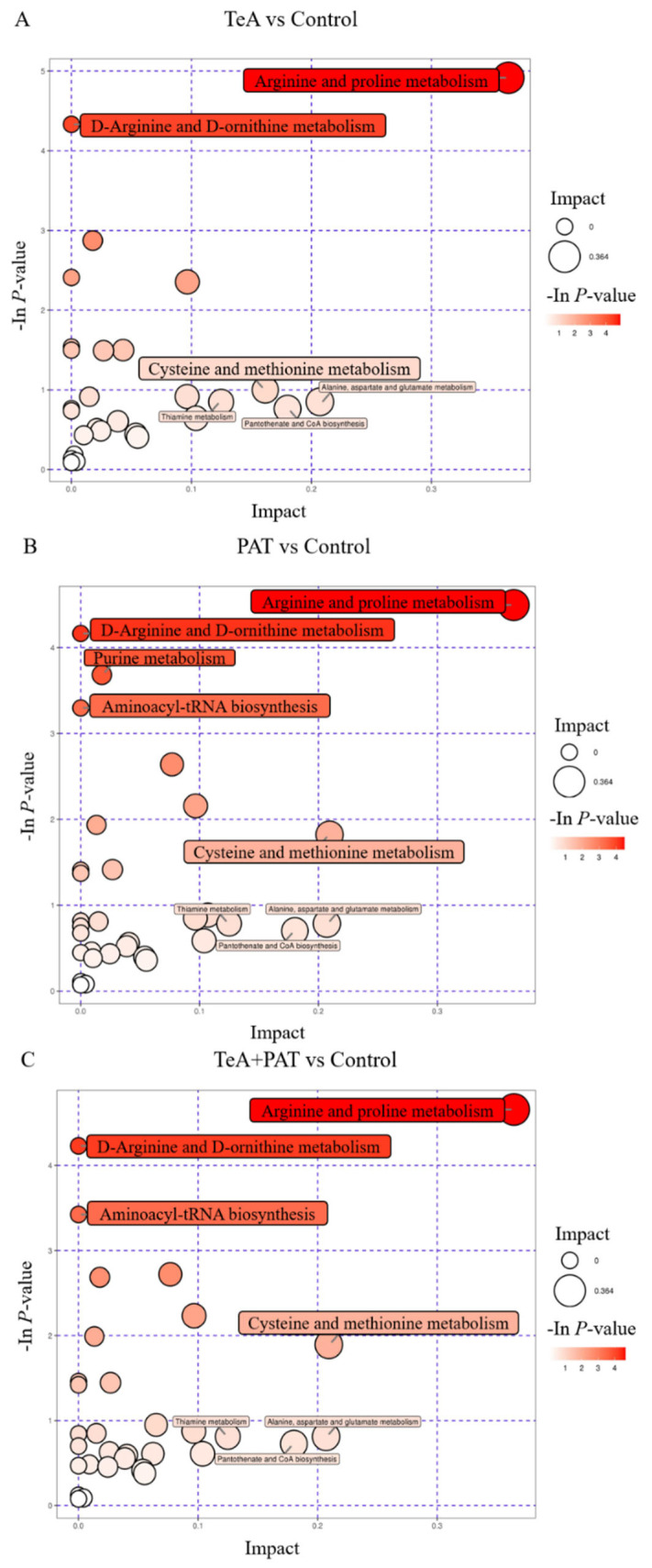
The metabolism pathway bubble plot of Caco-2 cells treated with TeA (**A**), PAT (**B**), and TeA + PAT (**C**). The x-coordinate and bubble size represent the influence factor size of the pathway in the topological analysis, and the y-coordinate and bubble color represent the *p*-value of the enrichment analysis. The larger the circle is, the greater the influence value is, and the deeper the color is, the more significant the result is.

Complex metabolic reactions and regulations in organisms are not carried out alone but rather form complex pathways and networks of different genes and proteins whose mutual influences and regulations can cause systemic changes in the metabolome. To further analyze the effects of TeA, PAT, and TeA + PAT exposure on metabolic pathways in Caco-2 cells, the SDMs of each experimental group were imported into the KEGG Pathway database for enrichment analysis and topological analysis, so as to discover the key pathways affecting the corresponding toxicity. As shown in Table 2, 28 metabolic pathways were affected by TeA exposure alone, including 14 amino acid metabolism pathways, 4 vitamin and cofactor metabolism pathways, 3 lipid metabolism pathways, 3 carbon hydrate metabolism pathways, 2 nucleotide metabolism pathways, 1 energy metabolism pathway, and 1 genetic information translation pathway. For the PAT-exposed group, 32 metabolic pathways were affected, that is, 15 amino acid metabolism pathways, 5 vitamin and cofactor metabolism pathways, 5 lipid metabolism pathways, 3 carbon hydrate metabolism pathways, 2 nucleotide metabolism pathways, 1 energy metabolism pathway, and 1 genetic information translation pathway. In the co-exposure of the TeA + PAT pattern, there were 32 metabolic pathways affected in total, that is, 14 amino acid metabolism pathways, 6 carbon hydrate metabolism pathways, 4 lipid metabolism pathways, 4 vitamin and cofactor metabolism pathways, 2 nucleotide metabolism pathways, 1 energy metabolism pathway, and 1 genetic information translation pathway. Moreover, further comparative analyses showed that there were 28 identical metabolic pathways and 4 different metabolic pathways (valine, leucine, and isoleucine degradation; ether lipid metabolism; sphingolipid metabolism; and riboflavin metabolism) between TeA and PAT groups, 27 identical metabolic pathways and 5 different metabolic pathways (valine, leucine, and isoleucine degradation; ether lipid metabolism; riboflavin metabolism; glycolysis/gluconeogenesis; citrate/TCA cycle; and pyruvate metabolism) between TeA and TeA + PAT groups, and 31 identical metabolic pathways and 3 different metabolic pathways (glycolysis/gluconeogenesis; citrate/TCA cycle; and pyruvate metabolism) between PAT and TeA + PAT groups. Meanwhile, there were 25 identical metabolic pathways among TeA, PAT, and TeA + PAT groups, including 12 amino acid metabolic pathways, 3 lipid metabolic pathways, 4 vitamin and cofactor metabolic pathways, 2 carbohydrate metabolic pathways, 2 nucleotide metabolic pathways, 1 energy metabolic pathway, and 1 genetic translation metabolic pathway. Among all the above-mentioned metabolic pathways, arginine and proline metabolism was the most important metabolic pathway with the highest enrichment degree and the largest influence factor, followed by the pathway of D-arginine and D-ornithine metabolism (Figure 5). Importantly, the pathways of cysteine and methionine metabolism and aminoacyl-tRNA biosynthesis were obviously enhanced in the group of TeA + PAT (Figure 5).

### 2.5. Validation of Key Metabolic Pathways

**Figure 6:**
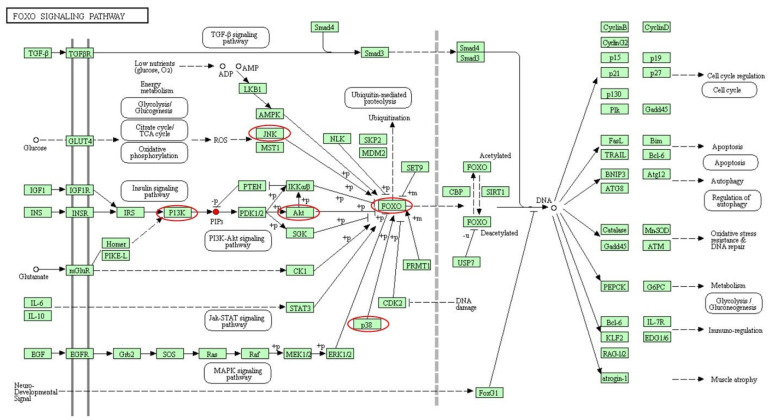
Changes in metabolites in PI3K/AKT/FOXO signaling pathway after TeA, PAT alone, and combined exposure. Red dot represents the metabolite that was detected. Red circles represent the genes that were selected to be verified.

**Figure 7:**
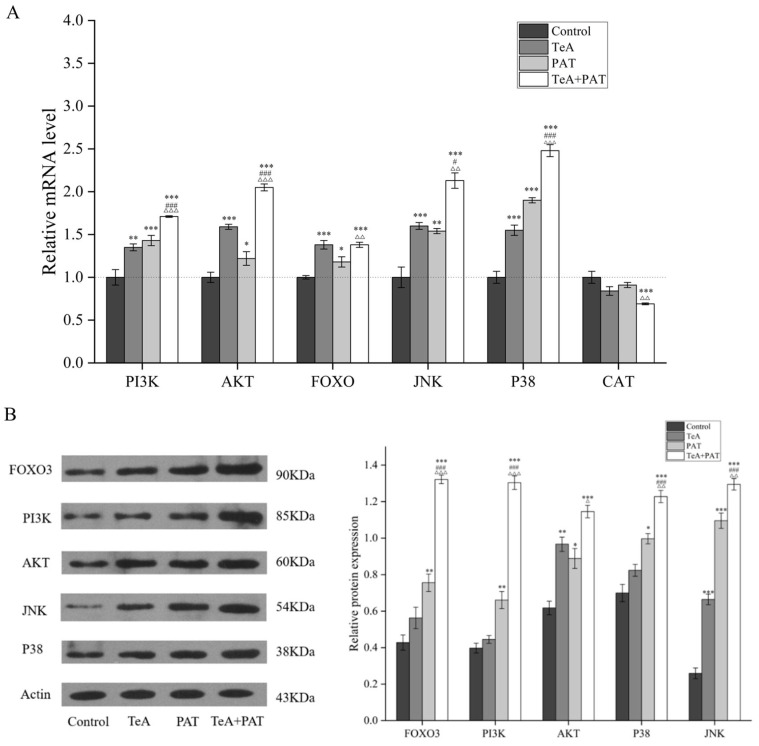
Effects of TeA, PAT, and TeA + PAT exposure on the expression levels of key genes (**A**) and the corresponding proteins (**B**). Data are expressed as mean values ± SD of independent experiments (*n* = 3). *, **, and *** represent significant difference (*p* < 0.05) or extremely significant difference (*p* < 0.01, *p* < 0.001) from the control group; ^#^, ^###^ and ^∆^, ^∆∆^, ^∆∆∆^ represent significant difference (*p* < 0.05) or extremely significant difference (*p* < 0.001) from TeA- and PAT-alone exposure groups, respectively.

Based on the metabolomics outcomes integrated with KEGG pathway analysis, phosphatidylinositol triphosphate (PIP_3_), as the significant intermediate of the PI3K/AKT/FOXO signaling pathway, was screened out in our study (Figure 6). The contents of PIP_3_ in TeA, PAT, and TeA + PAT groups all increased (*p* < 0.05) with FC values of 1.59, 1.55, and 1.72, respectively, indicating that TeA and PAT may have a similar toxic mechanism of action and the pathway of PI3K/AKT/FOXO may play an important role in the combined toxicity induced by TeA and PAT. Therefore, to confirm our hypothesis, the expressions of some key genes related to the PI3K/AKT/FOXO signaling pathway (pi3k, akt, foxo, jnk, p38, and cat) were subsequently quantified (Figure 7). Moreover, MAPK is the upstream signaling pathway of FOXO and is involved in important reactions, such as oxidative stress, and CAT is a downstream signaling molecule of FOXO, which can reflect the oxidative stress level of the body [29]. As shown in Figure 7A, the expression levels of pi3k, akt, foxo, jnk, and p38 were significantly up-regulated (*p* < 0.05) with the expression levels of 1.35-/1.43-/1.72-, 1.59-/1.22-/2.05-, 1.38-/1.18-/1.47-, 1.60-/1.54-/2.13-, and 1.55-/1.90-/2.48-fold compared with the control, respectively, corresponding to the TeA, PAT, and TeA + PAT experimental groups. Notably, the expression levels of these genes mentioned above significantly increased (*p* < 0.05) in the TeA + PAT group when compared with the TeA or PAT groups, hinting that PI3K/AKT/FOXO, p38 MAPK, and JNK signaling pathways were involved in the synergistic toxicity induced by the co-exposure of TeA and PAT. In addition, the expression of cat was significantly down-regulated in all experimental groups, among which the TeA + PAT group revealed the most significant down-regulation (*p* < 0.05). This result was consistent with the previous determination of CAT activity in Figure 2B. Next, the expressions of related proteins were determined using WB (Figure 7B). This displayed that the levels of AKT and JNK proteins of the TeA group and PI3K, AKT, P38, JNK, and FOXO3 proteins of the PAT group were significantly increased (*p* < 0.05) and that the levels of PI3K, AKT, P38, JNK, and FOXO3 proteins of the TeA + PAT group were not only significantly increased (*p* < 0.001) but also significantly higher than those in the TeA or PAT groups (*p* < 0.05).

## 3. Discussion

Both TeA and PAT are major pollutants with high detection rates in fruits, vegetables, nuts, and their products, and the detection rate of TeA in baby food can be as high as 96.51% [5,8,9,11,13]. Meanwhile, many studies have also found the coexistence of TeA and PAT, with a coexistence rate in apples as high as 46.43% [10,17,18]. Once ingested, the gut, as the first organ of contact after oral exposure, may face more serious challenges than other organs. Therefore, it is of great significance to investigate their combined toxic effects and mechanisms. In this study, compared to a single exposure to TeA or PAT, higher cell mortality rates of Caco-2 cells were observed under co-exposure to the mixture of TeA + PAT in a dose–response manner and showed that combined contamination could lead to more serious toxic effects, which should be paid more attention in the future.

It is universally acknowledged that a mass of free radicals could be produced when the body is exposed to exogenous toxic chemicals, which may put the body in a state of oxidative stress if the content of free radicals exceeds the body’s scavenging capability. ROS, as the main reactive substance that causes oxidative stress damage in cells, was found to be significantly increased in the TeA + PAT group, indicating the REDOX balance may be broken. This was also consistent with the determination of a significant reduction in CAT content shown in Figure 2B. Furthermore, mitochondria, as the energy metabolism center, can produce ATP as well as some byproducts, like ROS, under normal conditions; however, excessive ROS could be produced to attack mitochondria and cause mitochondrial dysfunction once attacked by some exogenous hazards [30]. This could partially explain the decrease in ATP content, especially in the TeA + PAT group. Similarly, it also was reported that the combination of aflatoxin B_1_ (AFB_1_) and deoxynivalenol (DON) can aggravate ROS production and reduce ATP levels by inducing abnormal expressions of Caspase-3, Bax, and Bcl-2 proteins through the mitochondrial pathway to produce synergistic toxicity in HepG2/C3A cells [31]. Similarly, a study has shown that co-exposure to PAT and citrinin (CTN) results in synergistic effects and increased ROS production in H-SY5Y cells [32]. It was reported that combined exposure of Alternaria alternata toxins (AOH, AME, and TeA) to GES-1 cells has a synergistic effect by activating caspase-3 cleavage to participate in the mitochondria-dependent apoptosis process and the activation of DNA damage signaling pathways ATR-Chk1-P53 and ATM-Chk2-P53 [15]. Moreover, it has been proved that the cytotoxicity of PAT is also mediated by the mitochondrial pathway [29]. Thus, it is speculated that the synergistic toxicity of TeA + PAT co-exposure may be caused by the excessive ROS accumulation, leading to oxidative stress and the inhibition of ATP production, in turn resulting in disorders of energy metabolism, but the relevant mechanisms still need to be further studied.

Since all cellular components, including lipids, proteins, nucleic acids, and sugars, can be damaged by oxygen free radicals, metabonomics was chosen in our study to mine the effects of exposure to TeA, PAT, and TeA + PAT, respectively, on Caco-2 cells. The results showed that the metabolic profiles of all the experimental groups were significantly affected, among which glycerophospholipids, amino acids, and a few aromatic compounds, fatty acids, sphingolipids, quinones, and terpenoids were significantly changed. Glycerophospholipid, as a structural component of the cell membrane, is ubiquitous in tissues, and usually participates in cell metabolism and protein signal transduction to regulate cell function [33]. In addition to causing lipid peroxidation, oxidative stress can also cause oxidative damage to proteins. Since many proteins are enzymatic proteins with catalytic functions, alterations in these proteins (amino acids) may have amplification effects. Several amino acids associated with the function of proteins, such as arginine, proline, cysteine, tryptophan, etc., are particularly sensitive to free radical damage. Importantly, arginine not only participates in the TCA cycle to regulate energy metabolism but is also the precursor for synthesizing a variety of bioactive substances, like polyamines, NO, etc. For instance, it can be decomposed into ornithine and inosinic acid by amidotransferase, and then ornithine may further generate polyamines, which play an important role in the regulation of cell growth and development [34]. Proline, synthesized from arginine or glutamate, is involved in cell proliferation and differentiation, protein synthesis, and antioxidation, playing an important role in maintaining the normal physiological function of cells [35].

Furthermore, amino acids also are very important substances in the process of energy metabolism [36]. From the metabolomics results (Table 2, Figure 5), arginine and proline metabolism were the metabolic pathways most affected by TeA, PAT, and TeA + PAT treatment, respectively, with the highest enrichment and the largest influence factors, indicating these two pathways should be the main approaches, as TeA and PAT affected cellular metabolism and exerted the corresponding toxic effects. Moreover, three unique metabolic pathways were unveiled after co-exposure of TeA + PAT as well, namely the TCA cycle, glycolysis/gluconeogenesis, and pyruvate metabolism. The TCA cycle is a common pathway of fat, amino acids, and protein metabolism. Since glycerophospholipid and amino acid metabolism were the most seriously affected by TeA + PAT, it is speculated that glycerophospholipid and amino acids may be converted into each other through the TCA cycle, thereby participating in the synergistic toxic effects induced by the co-exposure of TeA + PAT. It has been reported that the combined exposure of AFB_1_ and AFM_1_ induces a more extensive metabolic disorder in NCM460 cells, in which glycerophospholipid metabolism, fatty acid degradation, and propionic acid metabolism are the main pathways [37]. In addition, pyruvate, which always plays a key role in carbohydrate, lipid, amino acid, and energy metabolisms, is not only an important substance in the glycolysis/gluconeogenesis metabolic pathway but can also be oxidized to produce acetyl CoA in the TCA cycle. Meanwhile, many intermediate metabolites in the TCA cycle that contain ketone carbonyls (e.g., pyruvate) are capable of scavenging ROS to exert antioxidant effects [38]. Thus, combined with the above endpoints of oxidative stress, it could be conjectured that the co-exposure of TeA + PAT may cause synergistic toxicity by disturbing glycerophospholipids, arginine and proline metabolisms, the TCA cycle, glycolysis/gluconeogenesis, pyruvate metabolism, and the REDOX balance to affect normal cellular functions and metabolisms. Similar results were also obtained by another study showing that the combinations of DON + AFB_1_ and DON + ZEN + AFB_1_ could induce synergistic effects in Caco-2 cells by disrupting the amino acid metabolic pathways of glycine, serine, serine, and pyruvate [39]. It is worth mentioning that aminoacyl-tRNA biosynthesis was not among the highly enriched pathways with a greater influence in the TeA-treated group, but those in the TeA + PAT-treated group were notably promoted compared with those in the PAT-treated group. This result was consistent with a previous finding that PAT can inhibit the aminoacylation process by irreversibly inactivating aminoacyl-tRNA synthetases, suggesting that the perturbance of protein synthesis might also be one of the reasons for the synergistic toxicity [40].

**Figure 8:**
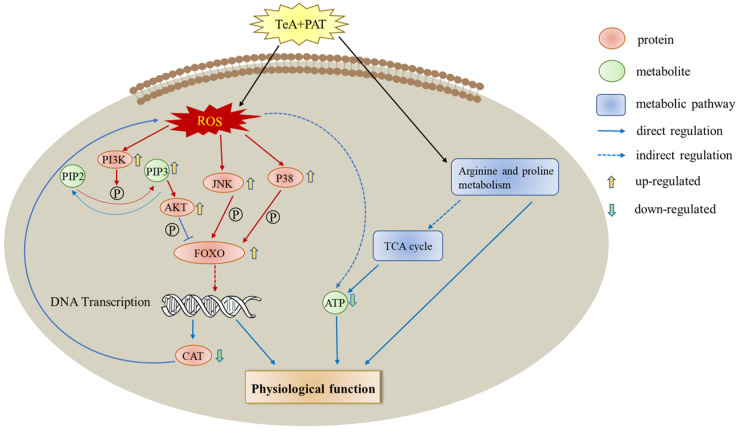
The main mechanisms of the synergistic toxic effects induced by the co-exposure of TeA and PAT.

Notably, PIP_3_ was found among the mass of metabolites, demonstrating that the PI3K-AKT pathway may be involved in the toxic effects produced by TeA and PAT and the synergistic effects of their mixture. The PI3K-AKT pathway is an essential intracellular signaling pathway associated with various cellular processes, such as cell differentiation, metabolism, inflammation, cell motility, cancer, etc. PI3K, as an important signal transduction molecule in cells, can catalyze the phosphorylation of phosphatid ylinositol-4,5-bisphosphate (PIP_2_) at the 3-hydroxy position to form PIP_3_, which acts as a second messenger to recruit 3-phosphoinositide-dependent protein kinase 1 (PDK 1) and serine/threonine kinase 3 (AKT) onto the cell membrane. At this point, PDK1 phosphorylates threonine located on AKT on the cell membrane, and then AKT is activated to subsequently phosphorylate its downstream targets, which finally could affect the normal signal transduction of cells [41]. PI3K can be activated by various types of cellular stimuli or toxic insults, including copper and zinc ions, growth factors, cytokines, and hormones, while AKT, as a growth factor-regulated serine/threonine kinase, is a key downstream target of PI3K [42]. The downstream signaling nodes of AKT, including FOXO, GSK3, and mTORC1, have been studied for many years, among which FOXO is one of the best-characterized transcription factors and plays an important role in maintaining homeostasis and adapting to environmental changes [43]. Research reported that FOXO1 was activated by ROS and then regulated by phosphatidylinositol 3-kinase/protein kinase B (P13K-PKB/Akt) [44,45]. Similarly, according to the PCR and WB results in Figure 7, PI3K/AKT/FOXO should be one of the core signaling pathways regulating the toxicity of TeA, PAT, and TeA + PAT. In addition, p38 mitogen-activated protein kinases (p38 MAPK) and c-Jun N-terminal kinase (JNK) signaling pathways are important upstream signaling pathways of FOXO, which has been proven to be important pathways involved in promoting cell apoptosis and death, regulating cell stress response, and so forth [46]. Under stress conditions, p38 and JNK can also be activated to affect the expression and function of downstream genes through a series of cascade phosphorylation reactions on FOXO, which strongly supports our results shown in Figure 7 [47]. Previous studies have also found that 15-acetyldeoxynivalenol (15ADON) and deoxynivalenol (DON) act jointly on GES-1 cells to activate the JNK and p38 pathways, and then the activation of JNK further promotes FOXO3a nuclear translocation, leading to apoptosis [48]. Moreover, the co-exposure of TeA and PAT increased the gene and protein expression levels of PI3K, AKT, P38, JNK, and FOXO when compared with TeA or PAT exposure alone, which was also consistent with the above-mentioned physiological and biochemical endpoints of Caco-2 cells in the current study. Similarly, researchers also reported that co-exposure to some foodborne chemicals can enhance toxic effects through the JNK/MAPK signaling pathway or inhibit toxicity by using certain bioactive substances through the AKT/FOXO/MAPK signaling pathway [49,50]. Overall, it could be concluded that the synergistic toxic effect of TeA + PAT in Caco-2 cells was mainly associated with the PI3K/AKT/FOXO signaling pathway together with the P38 MAPK and JNK signaling pathways (Figure 8).

## 4. Conclusions

To summarize, the co-exposure of TeA and PAT could induce synergistic toxic effects and enhance the oxidative stress response of Caco-2 cells and produce excessive ROS, which could act as signal molecules to activate PI3K/AKT and MAPK (P38, JNK) signaling pathways and finally act together on FOXO to interfere with the expression of downstream genes. At the same time, it also can directly affect arginine/proline metabolism, thereby regulating cell growth and development or inhibiting ATP production through the TCA cycle, which could result in abnormal energy metabolisms and affect the normal physiological function of Caco-2 cells.

## 5. Materials and Methods

### 5.1. Mycotoxins Solutions Preparation

TeA (purity ≥ 99%) and PAT (purity ≥ 99%) were purchased from Pribolab (Qingdao, China) and their stock solutions were prepared in DMSO at the concentrations of 20 and 10 μg/mL (stock solutions), respectively, stored at −20 °C in the dark. Working solutions were diluted with Dulbecco’s modified eagle’s medium (DMEM) supplemented with 10% fetal bovine serum and 1% antibiotic/antimycotic (penicillin and streptomycin). The above agents used in cell culture were obtained from Beijing Dingguo Changsheng Biotechnology, China. The final concentration of solvents (*v*/*v* = 0.1%) used for dissolving mycotoxins in the exposure medium had no adverse effect on the cellular parameters tested. Comprehensively considering the limit standard or suggested limit, the IC_50_ value of each mycotoxin obtained by a previous experiment, as well as the coefficient of uncertainty, and six incremental working concentrations of mycotoxins were prepared by two dilutions of the highest working concentration at 50 μg/mL for TeA and 2 μg/mL for PAT.

### 5.2. Cell Culture and Treatment for Cytotoxic Determination

Caco-2 cells, purchased from the Cell Bank of the Chinese Academy of Sciences (Shanghai, China), were grown in DMEM supplemented with 10% FBS and antibiotics in 25 cm^2^ cell culture flasks (Greiner, Monroe, NC, USA) at 37 °C in a humidified atmosphere of 5% CO_2_. The cells were left to reach a confluence of 80–90% and subsequently treated with 0.05% trypsin at three- or four-day intervals, while being suspended in Dulbecco’s modified eagle’s medium (DMEM) in a 1:3 split ratio. The passage of a batch of experimental cells was controlled within 10 generations. Cells were cultured in flasks for three days and then collected to seed in 96-well plates (Greiner, Monroe, NC, USA) with the optimum cell concentration of 1 × 10^5^ cells/well in 100 μL of the medium. Cells were allowed to grow and attach to the wells for 24 h before treatment with mycotoxins. Thereafter, the culture medium was replaced by a fresh medium containing working concentrations of each mycotoxin and their mixtures (same working concentrations). Parallel to the exposed group, untreated cells were taken as the control group, which was set up in all experiments. Based on the results of pilot studies showing that the inhibitory effect and differential responses of mycotoxins on cell viability were most obvious at 48 h in comparison to 24 or 72 h, the old medium was removed and replaced by 100 μL fresh medium containing 10% CCK-8 reagent (GLPBIO, Montclair, CA, USA) after incubation of another 48 h, and then the plates were incubated for another 4 h. Subsequently, plates were read by a multifunctional microplate reader (Bio-Rad, Hercules, CA, USA) to measure the absorbance (OD value) at 450 nm, which can indirectly reflect the number of viable cells. The cellular viability can be calculated by the following general equation:

Viability(%) = [OD_(treatment)_ − OD_(blank)_]/[OD_(control)_ − OD_(blank)_] × %100% (1)

where OD is the optical density for measuring the absorbance of each experimental group.

### 5.3. Determination of Reactive Oxygen Species (ROS) Level

DCFH-DA (Sigma-Aldrich Inc., St. Louis, MO, USA), short for 2′,7′-dichlorofluorescin diacetate, was used to detect the intracellular level of hydrogen peroxide (H_2_O_2_) to measure the level of ROS. Cells with a density of 2 × 10^5^ cells/mL were transferred to each well (1 mL) of a 12-well plate to allow the cells to grow and attach to the wells for 24 h. Then, freshly prepared working solutions, including the blank solutions were added to each well, respectively. After 48 h of exposure, the appropriate amount of DCFH-DA (10 μmol/L) was added to each well and cultured for 1 h in the dark after removing the old medium. Then, the treated cells were washed with PBS (Beijing Dingguo Changsheng Biotechnology, Beijing, China) 3 times to remove excessive fluorescent probes, followed by being quickly placed under a fluorescence microscope (OLYMPUS, Hachioji-shi, Japan) to take a record.

### 5.4. Determination of Catalase (CAT) Activity

CAT activity was detected according to the manufacturer’s protocol for the corresponding assay kits (Beyotime Biotechnology, Shanghai, China). In brief, adequate samples were prepared in advance and added to the appropriate amount of trypsin/EDTA solution (Beijing Dingguo Changsheng Biotechnology, Beijing China). Afterward, the supernatants were isolated after being centrifuged at 10,000 rpm for 5 min at 4 °C. Then, 20 μL of the supernatant of each sample was mixed with 20 μL of the catalase detection buffer and 10 μL of the hydrogen peroxide solution to react at 25 °C for 5 min. Right after that, 450 μL of the catalase reaction termination solution was added to each sample to terminate the reaction. Subsequently, 10 μL of the sample solution and 40 μL of the catalase detection buffer were added to a new 1.5 mL centrifuge tube and then mixed well. Finally, the above-prepared solutions to be tested (10 μL/well) and the chromogenic solution (200 μL/well) were added to a 96-well plate for incubation for 15 min at 25 °C to measure the corresponding absorbance (A = 520 nm).

### 5.5. Determination of Adenosine-Triphosphate (ATP) Level

Supernatants were obtained as described regarding the determination of CAT activity and then detected according to the manufacturer’s protocol of the corresponding assay kits (Beyotime Biotechnology, Shanghai, China). Briefly, 20 μL supernatants and 100 μL ATP detection working solutions were loaded into a 96-well black plate and mixed well, followed by measuring the corresponding relative light unit (RLU) value using a multifunctional microplate reader (Bio-Rad, Hercules, CA, USA). Afterward, the ATP value of each sample was calculated based on the ATP standard curve and the protein concentration detected by a bicinchoninic acid (BCA) assay, which was conducted using the routine protocol. The BCA assay was conducted by culturing the mixture of 20 μL of the supernatant and 200 μL of the BCA working solution in a 96-well plate at 37 °C for 30 min followed by detecting the corresponding absorbance at 520 nm. Finally, the measured ATP should be normalized to the protein concentration.

### 5.6. UHPLC-MS/MS Analysis for Cell Metabolomics

According to previous findings, 12.5 μg/mL of TeA, 0.5 μg/mL of PAT, and their mixture at the same concentrations were selected to treat Caco-2 cells for the following studies. The experimental groups and blank group were 12.5 μg/mL of TeA, 0.5 μg/mL of PAT, 12.5 + 0.5 μg/mL of TeA + PAT, and cell culture medium without FBS. The treated cells were removed from the cell medium, and the cells were quickly washed twice with precooled PBS before digestion with trypsin. Then, 5 volumes of precooled quencher (methanol/ammonium bicarbonate (85 g/L)/water = 6/1/3, pH = 7.4) were added to one volume of cells (containing 1 × 10^7^ cells) to be tested and then centrifuged at 1000× *g* at 4 °C for 1 min. Subsequently, the metabolites of the sample were extracted according to Doppler’s method with minor changes [51]. Briefly, the supernatant was removed, and the cells were mixed with 1 mL of the extract solution (methanol/acetonitrile/water = 2/2/1, *v*/*v*/*v*) containing the isotope-labeled internal standard mixture. Afterward, the mixtures were frozen in liquid nitrogen for 1 min, thawed, rotated with a vortex (QL-901, Kylin-Bell Lab Instruments Co., Ltd, Nantong, China) for 30 s in sequence (repeated 2~3 times), and underwent an ultrasound with an ultrasonic cell shredder (JY92-IIN, Ningbo Scientz Biotechnology Co., Ltd, Zhejiang, China) for 10 min at 4 °C. Then, all samples were incubated in a low-temperature incubator (MDF-40V528, Anhui Zhongke Duling Commercial Appliance Co., Ltd, Anhui, China) at −40 °C for 1 h, and finally centrifuged at 12,000× *g* at 4 °C for 15 min using a high-speed freeze centrifuge (Multiuge X3R, Thermo Fisher Technology (China) Co., Ltd, Shanghai, China). All the supernatants were collected for the metabolomics profile analysis, and an equal aliquot of supernatant form from the sample was pooled together to obtain a quality control (QC) sample. LC-MS/MS was performed using a UHPLC system (Vanquish, Thermo Fisher Scientific, Waltham, MA, USA) with a UPLC BEH Amide column (2.1 mm × 100 mm, 1.7 μm, Waters, MA, USA) coupled to the Q Exactive HFX mass spectrometer (Thermo Fisher Scientific, Waltham, MA, USA) and analyzed in both positive and negative ion modes. The mobile phase consisted of 25 mmol/L ammonium acetate and 25 mM/L ammonium hydroxide in water (pH = 9.75) (A) and acetonitrile (B). In total, 2 μL of diluent was applied to the column, and the elution gradient was set as follows: 0–0.5 min, 95% B; 0.5–7 min, 95–65% B; 7–8 min, 65–40% B; 8–9 min, 40% B; 9–9.1 min, 40–95% B; 9.1–12 min, 95% B. The temperatures used were 25 °C for the column and 4 °C for the autosampler. The QE HFX mass spectrometer was used to acquire MS/MS spectra on the information-dependent acquisition (IDA) mode in the control of the acquisition software Xcalibur (v4.3, Thermo Fisher Scientific, Waltham, MA, USA). The ESI source conditions were set as follows: a sheath gas flow rate of 30 Arb; an Aux gas flow rate of 25 Arb; a capillary temperature of 350 °C; a full MS resolution of 60,000; an MS/MS resolution of 7500; collision energy of 10/30/60 in NCE mode; and spray voltage of 3.6 kV (positive) or −3.2 kV (negative).

### 5.7. Real-Time Quantitative PCR (RT-qPCR)

The expression levels of the core genes involved in the key signaling pathways according to the metabolomic analysis were investigated. cDNA synthesis was carried out with total RNA extracted by Trizol^®^ Reagent (Invitrogen, Carlsbad, CA, USA) using the All-In-One 5×RT MasterMix Kit (Applied Biological Materials, Chongqing, China). RT-qPCR reactions were carried out by following the protocol of the NovoStart@SYBR qPCR SuperMix Plus Kit (Novoprotein, Chongqing, China) and then detected using the ABI StepOne Real-time PCR System (Thermo Fisher Scientific, Waltham, MA, USA). RT-qPCR was performed with a program of 40 cycles of 1 min at 95 °C, followed by 20 s at 95 °C and 1 min at 60 °C. The relative expression levels of the target genes were calculated using the 2^−ΔΔ CT^ method. Three technical replicates and three biological replicates were conducted. The target genes and their corresponding primers used in this study are listed in Table S1.

### 5.8. Western Blotting (WB) Assay

Previously treated cells were sonicated and centrifuged to collect the supernatants, which were boiled in boiling water for 10 min with five volumes of freshly prepared buffer. The protein concentrations of supernatants were determined using the method of bicinchoninic acid (BCA) protein assay, and then and the protein concentration of each group was adjusted to be consistent to obtain protein samples. Next, 20 μg of each sample was loaded on 12% sodium dodecylsulphate polyacrylamide gel electrophoresis (SDS-PAGE) running for 100 min at a constant voltage of 90 V and then transferred onto polyvinylidene difluoride (PVDF) membranes at the maximum voltage and 1.5 mA/cm^2^ of the gel area for 1.5 h. After blocking the nonspecific binding with skimmed milk (5% *w*/*w* fat content) for 1 h at room temperature, samples were incubated with diluted primary antibodies at 4 °C overnight followed by three sessions of washing (10 min/each). Subsequently, samples were incubated with diluted secondary antibodies for 2 h at 37 °C and then washed three times. Imaging exposure was carried out with Pierce ECL in the dark.

### 5.9. Statistical Analyses

All results were displayed as the mean ± standard deviation (SD). Data were analyzed by using the one-way ANOVA test and the Bonferroni test, and IC_50_ values were also obtained by probit regression in the SPSS statistics package, version 24 (SPSS, CHI, Chicago IL, USA). Figures were generated using Origin 2019 (Origin Lab, Northampton, MA, USA) and Image J 2022 (National Institutes of Health, Bethesda, MD, USA). *p* ≤ 0.01 and *p* ≤ 0.05 were considered extremely significant and significant regarding differences between experimental groups. The cell viability test and metabolome experiment were performed in six replicates, and the other experiments were performed in triplicates. Combined toxicity was evaluated by CompuSyn software 1.0 (https://www.combosyn.com/; accessed on 20 March 2022) based on the Chou–Talaly method [52]. The raw data of metabolomics were converted to the mzXML format using ProteoWizard and processed with an in-house program, which was developed using R and based on XCMS, for peak detection, extraction, alignment, and integration. Then, an in-house MS2 database (BiotreeDB) was applied for metabolite annotation. The cutoff for annotation was set at 0.3. The normalized metabolomic data were analyzed by principal component analysis (PCA), an unsupervised analysis that reduces the dimensions of the data, to visualize the distribution and the grouping of the samples. A 95% confidence interval in the PCA score plot was used as the threshold to identify potential outliers in the dataset. In order to visualize group separation and find significantly changed metabolites, supervised orthogonal projections to latent structures–discriminate analysis (OPLS-DA) was applied. Furthermore, the value of variable importance in the projection (VIP) of the first principal component in OPLS-DA analysis was obtained. The metabolites with VIP > 1 and *p* < 0.05 (Student’s *t*-test) were considered significantly changed metabolites. In addition, commercial databases including KEGG (http://www.genome.jp/kegg/; accessed on 18 May 2022) and MetaboAnalyst 5.0 (http://www.metaboanalyst.ca/; accessed on 25 May 2022) were used for pathway enrichment analysis.

## Figures and Tables

**Table 1:** Parameters and descriptions of combined toxicity assessment based on Chou–Talalay method.

Mycotoxin	Dose-Effect Parameters			CI Values and Descriptions					
	Dm/(μg/mL)	m	r	IC_25_		IC_50_		IC_75_	
TeA	32.46	1.28	0.99						
PAT	0.69	2.65	0.98						
TeA + PAT	10.21	2.58	0.96	1.03	Nearly addictive	0.87	Slightly synergism	0.77	Moderate synergism

**Table 2:** The metabolic pathway enrichment analysis of TeA-, PAT-, and TeA + PAT-treated Caco-2 cells.

KEGG Pathways	TeA	PAT	TeA + PAT
Amino acid metabolism	Arginine and proline metabolism; D-Arginine and D-ornithine metabolism; Glycine, serine and threonine metabolism; Glutathione metabolism; D-Glutamine and D-glutamate metabolism; Cysteine and methionine metabolism; Selenoamino acid metabolism; Alanine, aspartate and glutamate metabolism; Valine, leucine and isoleucine degradationvbeta-Alanine metabolism; Tryptophan metabolism; Histidine metabolis; Phenylalanine metabolism; Tyrosine metabolism	Arginine and proline metabolism; D-Arginine and D-ornithine metabolism; Glycine, serine and threonine metabolism; Glutathione metabolism; D-Glutamine and D-glutamate metabolism; Cysteine and methionine metabolism; Selenoamino acid metabolism; Alanine, aspartate and glutamate metabolism; Valine, leucine and isoleucine degradation; beta-Alanine metabolism; Tryptophan metabolism; Histidine metabolism; Phenylalanine metabolism; Tyrosine metabolism; Valine, leucine and isoleucine degradation	Arginine and proline metabolism; D-Arginine and D-ornithine metabolism; Glycine, serine and threonine metabolism; Glutathione metabolism; D-Glutamine and D-glutamate metabolism; Cysteine and methionine metabolism; Selenoamino acid metabolism; Alanine, aspartate and glutamate metabolism; Valine, leucine and isoleucine degradation; beta-Alanine metabolism; Tryptophan metabolism; Histidine metabolism; Tyrosine metabolism; Valine, leucine and isoleucine degradation
Lipid metabolism	Glycerophospholipid metabolism; Primary bile acid biosynthesis; Steroid hormone biosynthesis	Glycerophospholipid metabolism; Primary bile acid biosynthesis; Steroid hormone biosynthesis; Ether lipid metabolism; Sphingolipid metabolism	Glycerophospholipid metabolism; Primary bile acid biosynthesis; Steroid hormone biosynthesis; Ether lipid metabolism
Metabolism of cofactors and vitamins	Thiamine metabolism; Pantothenate and CoA biosynthesis; Vitamin B6 metabolism; Porphyrin and chlorophyll metabolism	Thiamine metabolism; Pantothenate and CoA biosynthesis; Vitamin B6 metabolism; Porphyrin and chlorophyll metabolism; Riboflavin metabolism	Thiamine metabolism; Pantothenate and CoA biosynthesis; Vitamin B6 metabolism; Porphyrin and chlorophyll metabolism
Carbohydrate metabolism	Inositol phosphate metabolism; Galactose metabolism; Amino sugar and nucleotide sugar metabolism	Inositol phosphate metabolism; Galactose metabolism; Amino sugar and nucleotide sugar metabolism	Inositol phosphate metabolism; Galactose metabolism; Amino sugar and nucleotide sugar metabolism; Citrate cycle (TCA cycle); Glycolysis or Gluconeogenesis; Pyruvate metabolism
Nucleotide metabolism	Pyrimidine metabolism; Purine metabolism	Pyrimidine metabolism; Purine metabolism	Pyrimidine metabolism; Purine metabolism
Energy metabolism	Nitrogen metabolism	Nitrogen metabolism	Nitrogen metabolism
Translation	Aminoacyl-tRNA biosynthesis	Aminoacyl-tRNA biosynthesis	Aminoacyl-tRNA biosynthesis

## Data Availability

The datasets generated for this study are available upon request from the corresponding author.

## Supplementary Materials

The following supporting information can be downloaded at: https://www.mdpi.com/article/10.3390/toxins16070319/s1, Table S1: RNA primer sequences for real-time quantitative PCR; Table S2: VIP values and fold changes in common altered metabolites after exposure to TeA, PAT, and TeA + PAT; Figure S1: Score scatter plot and permutation plot test of OPLS-DA models; Figure S2: The number (A) and classification (B) of different metabolites in Caco-2 cells exposed to TeA, PAT, and TeA + PAT, respectively.
