# Supplementary material for: Metabolome and Its Mechanism Profiling in the Synergistic Toxic Effects Induced by Co-Exposure of Tenuazonic Acid and Patulin in Caco-2 Cells
Source: Toxins (Basel). 2024 Jul 15;16(7):319. doi: 10.3390/toxins16070319 (PMC11281550; doi:10.3390/toxins16070319)
Supplement: Supplementary file 1 [file toxins-16-00319-s001.zip › toxins-3058855-supplementary.pdf]

Metabolome and Its Mechanism Profiling in the Synergistic Toxic Effects Induced by Co-Exposure of Tenuazonic Acid and Patulin in Caco-2 Cells

Table S1. RNA primer sequences for real-time quantitative PCR

Gene	Forward (5’~3’)	Reverse (5’~3’)
β-actin	GCTCTTGCCAATGGGGATCGC	CAGTTAGCGCCCAAAGGACCAG
FOXO	TGGCAAGCACAGAGTTGGATGAAG	CATATCAGTCAGCCGTGGCAGTTC
PI3K	AGACTGGAGGGAGGTGATGATGC	GGCTTAGGTGGCTTTGGTGGAAG
AKT	AGGAGATGGAAGTGGCGGTCAG	GCAGGATCTTCATGGCGTAGTAGC
P38	GATCCAGTTCCTCGTGTACCAGATG	AGTCTTCGTTCACAGCCAGGTTG
JNK	AACTCTTCCCAGATTCCCTCTTCCC	GATGCTGTAAGGCGTCGTCCAC
CAT	CTCAGGTGCGGGCATTCTATGTG	GGTGGACCTCAGTGAAGTTCTTGAC

Table S2. VIP values and fold changes of common altered metabolites after exposed to TeA, PAT and TeA+PAT.

Metabolism	TeA		PAT		TeA+PAT	
	VIP	FC	VIP	FC	VIP	FC
Enrofloxacin	1.08	0.92	1.20	0.92	-	-
2-Propanoylthiazole	1.80	0.75	1.17	0.81	-	-
Matsutakic acid A	1.36	0.73	1.11	0.82	-	-
Kanzonol K	1.39	0.71	1.30	0.81	-	-
2-Hydroxycinnamic acid	1.23	0.87	1.01	0.90	-	-
1-Isothiocyanato-3-phenylpropane	1.07	3.27	1.68	3.89	-	-
dIDP	1.04	2.00	1.15	2.26	-	-
Dioscoretine	1.06	2.21	1.13	1.74	-	-
Nicotine	1.34	3.06	1.57	1.98	-	-
2',3'-Dihydro-phytomenadione	1.30	3.75	1.05	2.10	-	-
PC(15:0/14:1(9Z))	1.29	5.61	1.16	4.67	-	-
(9E)-Valenciaxanthin	1.15	3.56	1.01	2.88	-	-
Montecristin	1.37	3.03	1.26	2.70	-	-
PI(18:1(11Z)/16:1(9Z))	1.42	4.12	1.30	3.20	-	-
PI(16:1(9Z)/16:0)	1.35	5.41	1.20	3.81	-	-
DG(22:6(4Z,7Z,10Z,13Z,16Z,19Z)/22:2(13Z,16Z)/0:0)	1.34	3.74	1.14	2.21	-	-
Solanidine	1.41	34.07	1.29	12.32	-	-
Setariol	1.36	8.62	1.11	3.24	-	-
PC(18:1(11Z)/20:1(11Z))	1.43	5.14	1.05	5.25	-	-
LysoPE(20:1(11Z)/0:0)	1.24	1.81	1.20	2.06	-	-
LysoPE(16:0/0:0)	1.25	2.33	1.15	2.40	-	-

Hexylamine	1.21	2.01	1.18	2.10	-	-
3,4-Dimethyl-1,2-cyclopentanedione	1.18	1.99	-	-	1.24	1.73
LysoPE(16:1(9Z)/0:0)	1.28	2.01	-	-	1.12	0.72
PS(18:1(9Z)/22:2(13Z,16Z))	1.36	46.47	-	-	1.34	41.01
5-Hydroxydantrolene	1.39	3.65	-	-	1.48	4.62
3beta,7alpha-Dihydroxy-5-cholestenoate	1.08	4.96	-	-	1.04	5.46
LysoPE(0:0/20:5(5Z,8Z,11Z,14Z,17Z))	1.43	2.67	-	-	1.10	1.75
stearoyl sphingomyelin	1.22	3.80	-	-	1.11	3.11
SM(d18:0/22:0)	1.09	2.35	-	-	1.05	2.22
PS(14:0/20:0)	1.11	9.31	-	-	1.08	10.86
LysoPE(0:0/20:4(5Z,8Z,11Z,14Z))	1.11	1.60	-	-	1.16	1.80
PA(20:0/22:4(7Z,10Z,13Z,16Z))	1.42	5.03	-	-	1.30	2.54
PC(20:1(11Z)/18:2(9Z,12Z))	1.45	3.45	-	-	1.24	1.74
PC(P-16:0/14:0)	1.10	3.47	-	-	1.26	6.67
Dopamine 3-O-sulfate	1.28	1.62	-	-	1.40	1.75
PS(18:2(9Z,12Z)/18:0)	1.02	0.72	-	-	1.02	0.77
Cellobiose	-	-	1.41	5.35	1.43	3.93
5'-Methylthioadenosine	-	-	1.43	9.51	1.36	5.78
4-Methoxybenzyl O-(2-sulfoglucoside)	-	-	1.42	5.41	1.44	4.03
Phosphorylcholine	-	-	1.13	29.27	1.15	28.80
Dimethyl dialkyl ammonium chloride	-	-	1.25	0.37	1.30	0.35
Glycerophosphocholine	-	-	1.40	8.28	1.43	7.87
Leucyl-Isoleucine	-	-	1.31	1.92	1.22	1.47
Nervonyl carnitine	-	-	1.21	1.80	1.00	1.51
L-Agaridoxin	-	-	1.06	0.50	1.35	0.30
Valyl-Phenylalanine	-	-	1.36	2.31	1.29	1.84
Norepinephrine	-	-	1.28	4.12	1.28	3.70
Phenylalanyl-Isoleucine	-	-	1.10	2.48	1.06	2.20
Threoninyl-Leucine	-	-	1.30	2.30	1.30	1.72
Oxolan-3-one	-	-	1.12	0.82	1.34	0.86
Valyl-Valine	-	-	1.27	1.87	1.28	1.64
2-O-(5,8,11,14,17-Eicosapentaenoyl)-1-O-hexadecylglycero-3-phosphocholine	-	-	1.13	12.34	1.26	50.99
Linoleamide	-	-	1.08	1.42	1.15	1.29
N,N'-Diacetylhydrazine	-	-	1.10	0.41	1.35	0.25
PC(22:2(13Z,16Z)/16:0)	-	-	1.14	2.57	1.15	2.76
2-O-p-Coumaroylhydroxycitric acid	-	-	1.39	2.28	1.34	2.02
N-Acetylserine	-	-	1.15	3.57	1.07	2.45
7-Ketodeoxycholic acid	-	-	1.02	1.72	1.09	2.10
Glucosylceramide (d18:1/20:0)	-	-	1.39	7.74	1.38	6.24
L-Isoleucine	-	-	1.31	1.92	1.06	2.20
Urolithin A-3-O-glucuronide	-	-	1.19	1.49	1.21	1.55
4'-O-Methylglucoliquiritigenin	-	-	1.22	3.42	1.37	2.78
Caffeic acid 3-O-glucuronide	-	-	1.42	3.41	1.42	2.88

Methionyl-Leucine	-	-	1.32	2.13	1.19	1.62
Morphiceptin	-	-	1.11	7.08	1.03	4.23
IDP	-	-	1.26	1.59	0.83	1.94
Glycitein 4'-O-glucuronide	-	-	1.46	1.75	1.25	1.52
PC(15:0/16:0)	1.54	10215.67	1.23	4767.70	1.51	11448.53
PE(P-18:0/22:4(7Z,10Z,13Z,16Z))	1.09	1884.64	1.48	2699.64	1.51	2756.23
Imidazolelactic acid	1.54	1249.56	1.50	1866.45	1.51	1169.95
3-(4-Hydroxy-3-methoxyphenyl)-1,2-propanediol 2-O-(galloyl-glucoside)	1.51	146.25	1.48	207.92	1.50	214.43
Acetylcysteine	1.38	65.95	1.37	81.27	1.37	63.57
2-O-(5,8,11,14,17-Eicosapentaenoyl)-1-O-hexadecylglycero-3-phosphocholine	1.47	19.50	1.13	12.34	1.26	50.99
PS(18:1(9Z)/22:2(13Z,16Z))	1.36	46.37	1.06	13.96	1.34	41.01
PC(18:0/P-18:0)	1.43	27.72	1.36	20.75	1.40	27.40
Sarmentosin	1.25	18.70	1.29	28.72	1.29	25.79
Soyacerebroside I	1.40	50.01	1.18	14.99	1.29	19.67
Lactosylcermide (d18:1/20:0)	1.31	9.70	1.26	12.10	1.32	19.41
PI(20:3(5Z,8Z,11Z)/16:0)	1.32	21.33	1.32	14.40	1.36	19.33
Diepomuricanin A	1.26	16.44	1.50	114.33	1.25	18.00
Polyoxyethylene dioleate	1.31	19.86	1.33	14.38	1.34	16.56
PC(P-18:1(11Z)/20:1(11Z))	1.45	11.15	1.35	7.23	1.44	14.36
PC(P-18:1(11Z)/20:2(11Z,14Z))	1.48	10.53	1.42	9.39	1.44	12.31
PG(16:0/16:0)	1.45	9.32	1.42	8.08	1.46	12.19
PC(P-18:1(11Z)/22:4(7Z,10Z,13Z,16Z))	1.48	7.89	1.42	8.25	1.45	11.81
Coenzyme Q9	1.51	8.24	1.45	7.67	1.48	11.36
Artemoin A	1.47	9.48	1.42	8.80	1.44	10.90
PI(20:2(11Z,14Z)/18:2(9Z,12Z))	1.48	8.12	1.41	7.99	1.44	10.78
PC(P-18:1(11Z)/22:5(4Z,7Z,10Z,13Z,16Z))	1.45	10.56	1.42	7.67	1.45	10.56
PI(22:3(10Z,13Z,16Z)/18:3(6Z,9Z,12Z))	1.22	8.71	1.11	7.41	1.19	10.54
Octadecylamine	1.17	9.92	1.16	11.01	1.16	10.41
Hovenoside I	1.45	11.07	1.40	9.14	1.41	10.23
PC(P-16:0/20:1(11Z))	1.51	9.39	1.44	8.27	1.45	9.35
Lucidenic acid B	1.47	7.62	1.44	7.41	1.44	8.39
PA(22:2(13Z,16Z)/20:1(11Z))	1.48	8.15	1.42	7.62	1.45	8.26
PC(16:0/14:0)	1.48	8.10	1.42	8.02	1.42	8.01

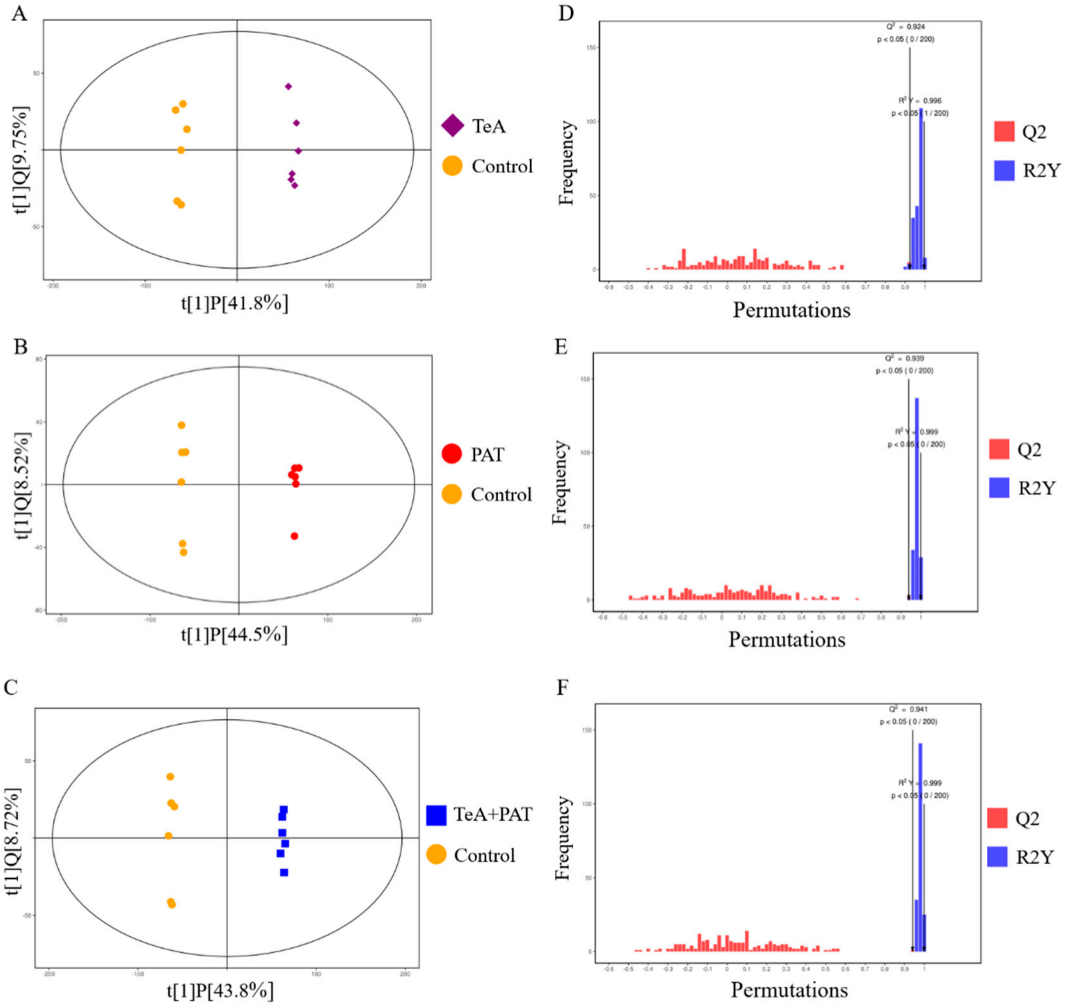

Figure S1. Score scatter plot and Permutation plot test of OPLS-DA models.

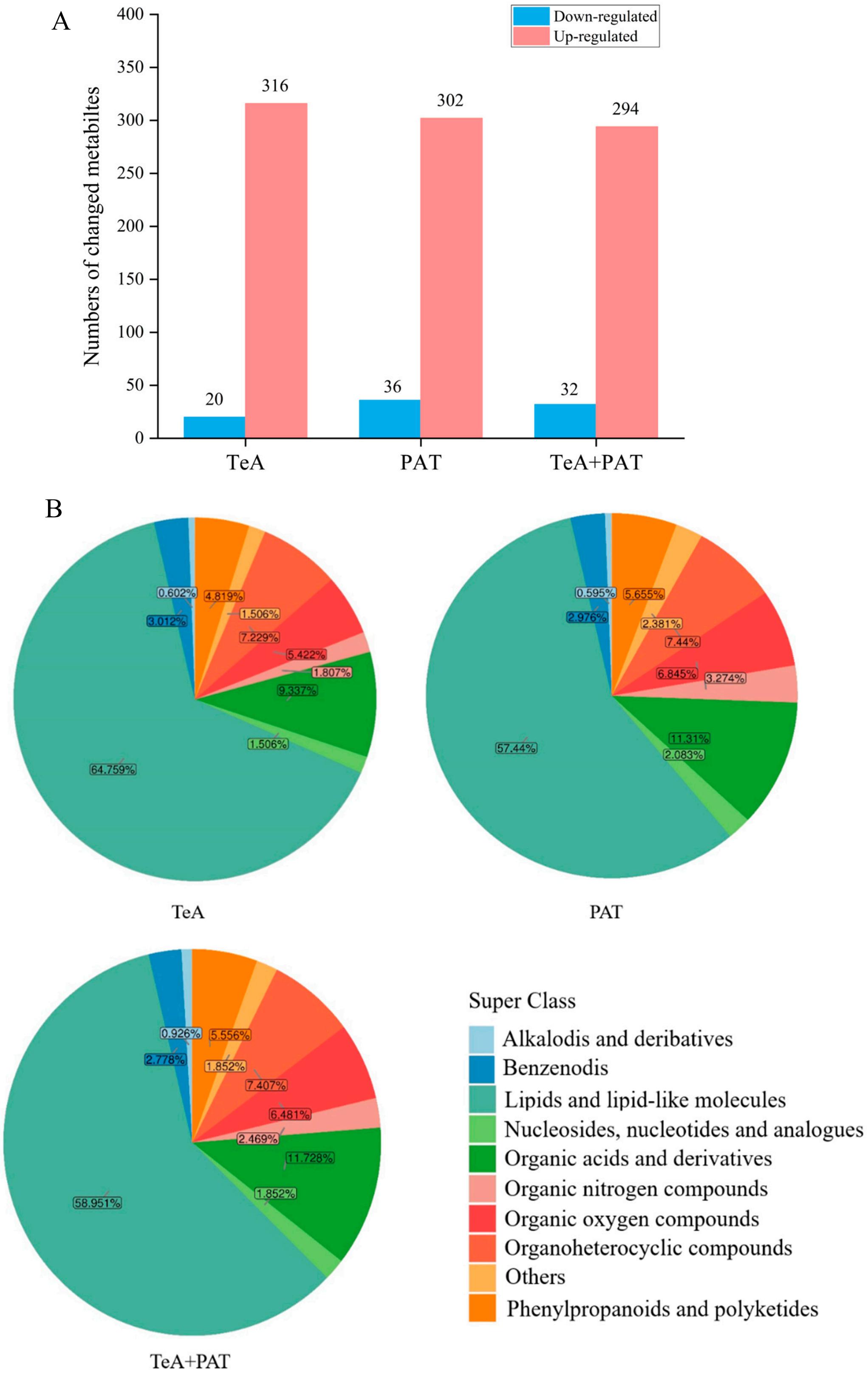

Figure S2. The number (A) and classification (B) of different metabolites in Caco-2 cells exposed to TeA, PAT and TeA+PAT, respectively.
